# Effects of a disposable home electro-stimulation device (Pelviva) for the treatment of female urinary incontinence: a randomised controlled trial

**DOI:** 10.1007/s00404-021-06179-4

**Published:** 2021-08-20

**Authors:** Jackie Oldham, Julia Herbert, Jane Garnett, Stephen A. Roberts

**Affiliations:** 1 Faculty of Biology, Medicine and Health, Manchester Academic Health Science Centre, University of Manchester, Manchester, UK; 2 Ellesmere Physiotherapy Clinic, Lancashire, UK; 3 JG Technology Management Ltd, Ipstones, UK; 4 Centre for Biostatistics, Faculty of Biology, Medicine and Health, Manchester Academic Health Science Centre, University of Manchester, Manchester, UK

**Keywords:** Electrostimulation device, Female urinary incontinence, Pelvic floor muscle, Randomised Controlled Trial, Rehabilitation

## Abstract

**Aims:**

To compare current General Medical Practitioner treatment as usual (TAU) for the treatment of female urinary incontinence with a novel disposable home electro-stimulation device (Pelviva).

**Methods:**

Open label, Primary Care post-market evaluation. 86 women with urinary incontinence were randomly assigned to one of two 12-week treatments: TAU or Pelviva for 30 min every other day plus TAU. Outcome measures included ICIQ-UI (primary), PISQ-IR, PGI-S / PGI-I and FSFI (secondary) at recruitment and immediately after intervention, 1-h pad test at recruitment and usage diaries throughout.

**Results:**

Pelviva plus TAU produced significantly better outcome than TAU alone: 3 versus 1 point for ICIQ-UI (Difference − 1.8 95% CI: − 3.5 to − 0.1, *P* = 0.033). Significant differences were also observed for PGI-I at both 6 weeks (*P* = 0.001) and 12 weeks (*P* < 0.001). In the Pelviva group, 17% of women described themselves as feeling very much better and 54% a little or much better compared to 0% and 15% in the TAU. Overall PISQ-IR score reached statistical significance (*P* = 0.032) seemingly related to impact (*P* = 0.027). No other outcome measures reached statistical significance. Premature termination due to COVID-19 meant only 86 women were recruited from a sample size of 264. TAU did not reflect NICE guidelines.

**Conclusions:**

This study suggests Pelviva is more successful than TAU in treating urinary incontinence in Primary Care. The study had reduced power due to early termination due to COVID-19 and suggests TAU does not follow NICE guidelines.

## Introduction

Bladder problems affect millions of people worldwide with 25–45% of women reporting some degree of urinary incontinence [1]. The impact can be considerable and distressing, affecting both quality of life and sexual function [2].

The most common types of incontinence in women are stress, urgency and mixed [3]. Stress urinary incontinence (SUI) is associated with leakage of urine during activities that increase intra-abdominal pressure (physical exertion, coughing, or sneezing). Urgency urinary incontinence (UUI) is associated with urgency, frequency or being woken at night to pass urine. Stress and urgency incontinence together is classified as mixed urinary incontinence (MUI).

Most conservative treatments target pelvic floor muscle function and include biofeedback, weighted cones and pelvic floor muscle exercises. Voluntary contraction of the pelvic floor is, however, fraught with difficulties with as many as 50% of women unable to correctly perform an unsupervised contraction [4–6].

NICE 2019 guidance recommend 3 months of supervised pelvic floor muscle exercises. However, urinary incontinence is a stigmatizing condition [7] and women do not tend to seek help [8], leaving them with little option other than to self-manage their problem. This highlights the importance of evidence-based self-management treatment options.

Pelviva is a commercially available single use, disposable Pelvic Floor muscle trainer, manufactured from compressible foam and is inserted like a tampon into the vagina and removed using a pull cord. It delivers effective stimulation to the pelvic floor muscles, eliciting a contraction as per the stimulation protocol described by Oldham et al. [9]. In a previous randomised controlled trial, Pelviva plus pelvic floor muscle exercise demonstrated a statistically significant (*P* = 0.014) improvement in quality of life as measured by the International Consultation on Incontinence Questionnaire – Urinary Incontinence Short Form (ICIQ-UI SF) compared to pelvic floor exercises alone in the treatment of all types of incontinence in women [9].

### Aims of the study

The trial by Oldham et al. in 2013 [9] was undertaken in a strict laboratory, highly controlled research environment and did not reflect the real world situation but suggested that Pelviva could offer an effective solution to the management urinary incontinence in Primary Care. This pragmatic trial was designed therefore to test the effectiveness of Pelviva in a real-world situation and to compare Pelviva with current General Medical Practitioner (GP) treatment for the management of female urinary incontinence.

### Primary hypothesis

A novel disposable home electro-stimulation device (Pelviva) is more effective than General Medical Practitioner treatment for female urinary incontinence in terms of frequency of urinary incontinence, amount of leakage and overall impact of urinary incontinence as measured by the ICIQ-UI SF.

## Materials and methods

An open-label, community-based, post-market, randomised controlled trial of a novel neuromuscular electrical stimulation treatment for urinary incontinence (Pelviva) was undertaken. Women with urinary incontinence (urgency, stress or mixed), determined through consultation with their General Medical Practitioner (GP), were randomised into one of two groups. The treatment as usual control group (TAU) received routine care, following each GP’s standard practice, for their urinary incontinence determined by their GP and may have included Pelvic floor exercises explained by the doctor/practice nurse; weighted exercise cones; pelvic toner device; vaginal insert device e.g. Contiform; pelvic floor physiotherapy; bladder re-training (Continence service); medication which could include anticholinergics or mirabegron. The intervention group received the Pelviva device in addition to active TAU. Treatment lasted for 12 weeks with a Quality of Life (QoL) primary end point immediately post-treatment.

### Participants and recruitment

Women aged between 18 and 65 years were recruited from practices within the South Manchester GP Federation. Subjects were identified by electronic health record searches (diagnosis, prescribed medication, treatment), contacted by text message from GPs and practice nurses and screened for eligibility by GPs.

Affirmative responders were sent a letter, including a participant information leaflet and sample consent form, followed by a telephone call inviting them to participate in the study. At the initial visit, women were individually consented and eligibility for participation in the study established. All participants were able to withdraw from the study via telephone, email or post at any time. All withdrawals were recorded electronically.

### Approvals and trial registration

Ethical approval was granted by Greater Manchester South Research Ethics Committee (REC Reference 17/NW/0395). MHRA approval was granted in 2009 (CI/2009/0008). Trial registration ClinicalTrials.gov identifier: NCT04059653.

### Inclusion and exclusion criteria

Inclusion criteria were women aged between 18 and 65, GP established urinary incontinence and willingness to participate in the study. GP determined exclusion criteria were: abnormal abdominal mass; clinical history of urinary retention problems: severe atrophic vaginitis, vaginal infection, vaginal lesion, severe urogenital prolapse at the level of the vaginal introitus or any other pathology of the vagina or labia; pregnancy or given birth within the last 3 months; implanted pacemaker; recent pelvic surgery (within the last 12 months); recent haemorrhage, haematoma and/or tissue damage to the vagina; undergoing any active therapy or review appointments for pelvic malignancy; manual dexterity insufficient to place the Pelviva device in the vagina; presence of a severe neurological conditions such as Multiple Sclerosis, Motor Neuron Disease or Parkinson’s Disease; multiple co-morbidities to the extent that the activities involved in the ‘pad test’ (i.e. stair climbing) cannot be completed; insufficient cognitive ability to provide informed consent and/or participate in the study. Any evidence of a urinary tract infection was confirmed by urinary dip stick test, with women treated accordingly and free of infection before being eligible to participate in the trial.

### Randomisation and blinding

Immediately after baseline assessment an online randomisation service (www.sealedenvelope.com) was accessed by the research team to assign women to either the Pelviva or TAU group utilising random permuted blocks with block sizes of 4–8. Participants could not be blinded to the treatment and were aware of the two potential treatment groups. The trial management, oversight and analysis team remained blinded to treatment allocation until after data lock.

### Intervention protocols

#### Control

As this trial was designed as a real world study the TAU group treatment was not pre-defined. As such, this could comprise of any of the treatments recommended in the NICE Clinical Guideline 123 [10], with the GPs free to select the most appropriate treatment for their patient including continuation of any medication that had already been prescribed.

#### Pelviva group

The Pelviva group continued with any existing prescribed GP care and in addition were prescribed a 12-week course of Pelviva treatment. Pelviva is a commercially available, single use disposable, fully automated, neuromuscular electrical stimulation device which is used every other day for 30 min. For further details, refer to Oldham et al. [9]. Any queries regarding use of the device were managed via telephone and email.

The stimulation programme was delivered using a duty cycle of 10-s stimulation followed by 10-s rest that runs for a period of 30 min. The Pelviva devices were pre-programmed to automatically gradually ramp-up the intensity of stimulation over a 24-s period to reach a therapeutic level and switch off automatically after 30 min. All devices were programmed to supply the same level of stimulation namely the average intensity that is considered comfortable and capable of producing a contraction of the pelvic floor muscles. This was determined through pilot safety and evaluation studies conducted in accordance with International Standards Organisation (ISO) 14,155. During the 10-s ‘‘on time’’, the device delivered a patent protected ten repeats of a short high-intensity burst of 50 Hz stimulation immediately preceded by a doublet (125 Hz), superimposed on continuous low-frequency 2-Hz stimulation. The devices will have an associated purchasing cost but it is envisaged this will be offset in part due to a reduction in use of other over the counter incontinence products.

### Outcome measures

#### Primary outcome measure

The primary outcome measure was the self-completed short form version of the International Consultation on Incontinence Questionnaire—Urinary Incontinence (ICIQ-UI-SF) [11]. Question items included frequency of urinary incontinence, amount of leakage, overall impact of urinary incontinence and an unscored self-diagnostic item. The 0–21 overall score (greater values indicating increased severity) was defined as the primary outcome. A clinically meaningful difference is described as a reduction of 2 or more points [11]. Subscales were specified as secondary outcomes.

#### Secondary outcomes

The secondary outcomes were included to assess the effects on sexual health, patient reported assessment of severity and improvement of their condition and an objective measure of amount of urine lost. These were assessment by utilisation of:

- The International Urogynaecology Association (IUGA) Pelvic Organ Prolapse/Urinary Incontinence Sexual Questionnaire (PISQ-IR) for evaluation of sexual dysfunction [12].
- The Patient Global Impression of Severity (PGI-S) and of Improvement (PGI-I) questionnaire for global assessment of improvement in incontinence [13].
- The Female Sexual Function Index (FSFI)—to assess impact on domains of sexual functioning (e.g. sexual arousal, orgasm, satisfaction, pain) [14].
- A 1-h in-clinic provocative pad weight test “1-h pad test” as defined by the International Continence Society (ICS) [15].

All questionnaires were collected immediately pre-treatment (at baseline), mid-treatment (at 6 weeks) and end of treatment (at 12 weeks). The 1-h pad test was planned at baseline and end of treatment but only completed at baseline due to COVID-19 restrictions.

Participants were also requested to complete diaries every other day for 4 weeks of the trial and then every week for the following 8 weeks to record treatment usage and usability specific to treatment arm.

Data for all questionnaires were collected electronically using a secure electronic data capture system Red Pill (https://www.sealedenvelope.com/redpill/). The system reminded participants to complete the questionnaire data in the privacy of their own home via mobile phone, tablet or computer and sent reminders for overdue questionnaires. Research nurses telephoned participants if multiple questionnaires or diaries were missing. Other case report form data (e.g. pad-test, eligibility criteria, urinalysis tests) were entered online by research nurses (Red Pill ePRO).

### Sample size

This study was initially powered to detect a 1.5-point mean difference (considered to be clinically meaningful) between groups in terms of quality of life immediately post-treatment as measured by the ICIQ-UI with conservative SD estimate of 3.75 based on a previous trial [9]. A sample size of 132 per group was determined (two-sided test, with 0.05 level of significance and 90% power). However, the study was terminated prematurely due to COVID-19 restrictions and this number was not achieved.

### Statistical analysis

The analysis was pre-specified in a Statistical Analysis Plan finalised prior to database lock and the unbinding of the Trial Statistician and Management Team.

Adherence to the active treatment was assessed by summing the patient reported number of devices used in each diary period, censoring the few that reported more devices used than prescribed at 100% and expressing this as a proportion of the number prescribed. As diary completion was incomplete, a second estimate conservatively assumed that missing diaries represented no usage. The analysis dataset included all women randomised who provided data for the primary outcome.

The primary analyses utilised standard analysis of covariance models adjusting for baseline and type of incontinence. Baseline, follow-up and within-patient changes were computed and summarised to aid interpretation. The four-point PGI-severity scale was dichotomised into normal/mild and moderate/severe and analysed using analogous logistic regression models. The ordinal PIG-I improvement measure was only meaningful at follow-up and the treatment arms were compared using a Mann–Whitney *U* test. All analyses were conducted in the R statistical environment with R-markdown scripts.

## Results

COVID-19 pandemic restrictions prevented face-to-face contact at the 12-week follow-up and the pad test could only be completed at baseline. Restrictions also led to premature closure of the trial and impacted the intention to undertake follow-up assessments annually for 3 years.

**Fig. 1:**
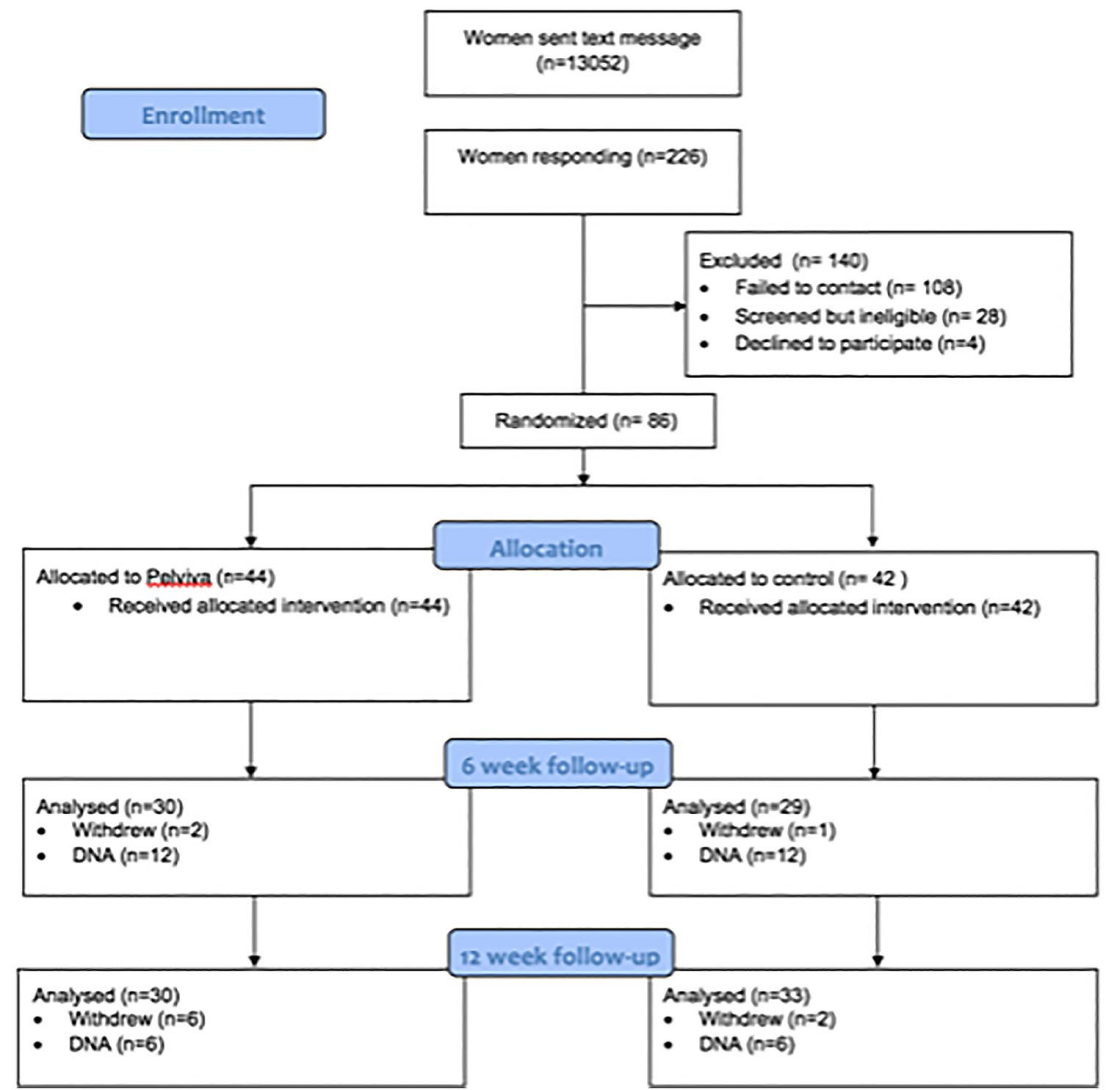
Consort diagram—summary of participant flow

The Consolidating Standards of Reporting Clinical Trials (CONSORT) diagram (Fig. 1) shows over a 12-week period 226 women responded to a total of 13,052 text messages (1.7%). Of these women, 108 could not be contacted and 4 withdrew before randomisation. A further 28 women were screened and found ineligible according to exclusion criteria.

Patient characteristics are summarised in Table 1. The majority of women had stress or mixed urinary incontinence, with only one describing standalone urgency. The majority of women (70%) demonstrated mild incontinence determined by urine loss on 1-h pad test as 10 g or less, 23% moderate between 10.1 and 50 g and 7% severe greater than 50 g loss.

**Table 1:** Patient characteristics

	All randomised		Analysis dataset	
	TAU	Pelviva	TAU	Pelviva
Type of incontinence				
Urgency	0/42 (0%)	1/44 (2%)	0/33 (0%)	1/30 (3%)
Stress	16/42 (38%)	11/44 (25%)	14/33 (42%)	9/30 (30%)
Mixed	26/42 (62%)	32/44 (73%)	19/33 (58%)	20/30 (67%)
Severity of incontinence (Pad test)				
Mild	31/42 (74%)	29/44 (66%)	26/33 (79%)	21/30 (70%)
Moderate	9/42 (21%)	11/44 (25%)	6/33 (18%)	7/30 (23%)
Severe	2/42 (5%)	4/44 (9%)	1/33 (3%)	2/30 (7%)
Age				
Median (IQR) {range}	51 (44–56) {32–66}	46 (41–55) {22–64}	51 (44–57) {32–66}	45 (41–55) {22–64}
Tampon User	26/28 (93%)	24/30 (80%)	24/26 (92%)	17/21 (81%)

Participant characteristics for all randomised women and women included in the primary analysis

At baseline 67% in the active TAU control group and 71% in the Pelviva group described no treatment for their incontinence. During the trial, the TAU group reported 31% receiving no active treatment compared to 68% in the Pelviva group. Of note 64% of women in the TAU group reported now doing unsupervised pelvic floor exercises, this increase was not observed in the Pelviva group.

Adherence to the Pelviva treatment was 78% based on the completed diary entries (devices used/devices prescribed). Not everyone fully completed the diary entries and if uncompleted dairies are conservatively assumed to represent no device usage, adherence was still 61%.

### Primary outcome: quality of life

**Fig. 2:**
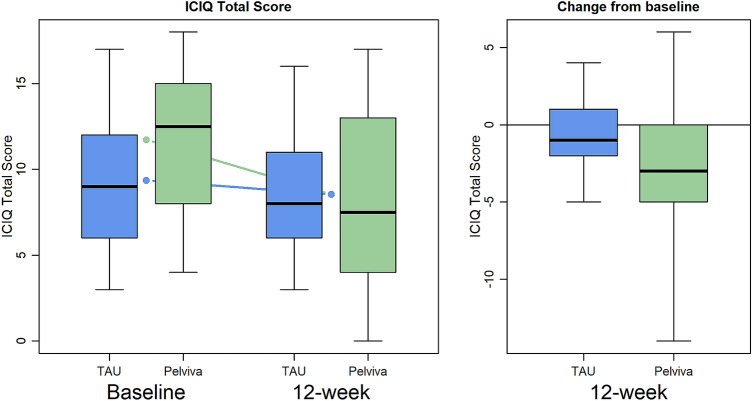
ICIQ-UI total score (primary outcome) at the two assessment times by treatment arm. Left hand panel shows the actual scores and the right-hand panel the changes from baseline. Boxplots represent the median, inter-quartile range and absolute range. Means per group are shown by points and lines

Results of the ICIQ-UI are summarised in Table 2 and Fig. 2.

**Table 2:** ICIQ-UI results

Outcome	Arm	Baseline	12-week follow-up	Change	Pelviva effect (95% CI)	*P*
ICIQ-UI	TAU (*N* = 33)	9.4 (3.9)	8.5 (3.8)	− 0.8 (2.1)		
	Pelviva (*N* = 30)	11.7 (4.2)	8.5 (4.9)	− 3.2 (4.2)	− 1.81 (− 3.49 − 0.14)	0.034
Frequency	TAU (*N* = 33)	2.5 (1.3)	2.4 (1.2)	− 0.1 (0.9)		
	Pelviva (*N* = 30)	3.0 (1.1)	2.3 (1.4)	− 0.7 (1.1)	− 0.36 (− 0.87 − 0.15)	0.17
Amount	TAU (*N* = 33)	2.5 (0.9)	2.4 (0.8)	− 0.1 (1.0)		
	Pelviva (*N* = 30)	3.0 (1.0)	2.3 (0.9)	− 0.7 (1.1)	− 0.24 (− 0.68 − 0.20)	0.28
Interference	TAU (*N* = 33)	4.4 (2.7)	3.8 (2.4)	− 0.6 (1.6)		
	Pelviva (*N* = 30)	5.7 (2.5)	3.9 (3.0)	− 1.9 (2.6)	− 0.91 ( − 1.97 − 0.15)	0.092

Results for ICIQ-UI total score (primary outcome) and subscales at the primary 12-week assessment point. Values are mean (SD). The effect size is the difference in outcome between arms adjusted for baseline and type of incontinence with 95% CI and associated significance level

After 12 weeks of treatment the Pelviva women showed a mean 3-point improvement compared to a mean < 1-point improvement for the control group on the 21-point scale, with an overall treatment effect of − 1.81 points (95% CI: − 3.49 to − 0.14, *P* = 0.033). There were no significant between group differences for frequency, amount and interference subscales, although the estimates of all the subscales did point to an improvement.

Improvements in overall ICIQ-UI were evident at 6 weeks with the Pelviva group with the treatment effect estimated as − 1.04 points (95% CI − 2.18 to − 0.10, *P* = 0.072).

### Secondary outcomes

#### Patient global assessment of severity and improvement

Given the early closure of the trial, there was little statistical power to detect changes in PGI-severity with the OR for moderate/severe symptoms being 0.5 (95% CI 0.1–1.6), With the OR for moderate/severe symptoms being 0.4 (95% CI 0.1–2.0), *P* = 0.26 at 6 weeks and 0.5 (95% CI 0.1–1.6), *P* = 0.22 at 12 weeks.

Statistically significant between group differences were observed for PGI improvement both at 6 weeks (*P* = 0.001) and 12 weeks (*P* < 0.001) (Table 3). 17% of women in the Pelviva group described themselves as feeling very much better and a further 54% a little or much better after 12 weeks compared to 0% and 15% of women in the control group. After 6 weeks, 52% of women in the Pelviva group were already feeling a little or much better compared to 14% in the control group. 3% of women in the Pelviva and 15% in the TAU groups described their condition as worsening.

**Table 3:** PGI results

	Mid-treatment *n* (%)	Post-treatment *n* (%)
Pelviva		
Very much better	1 (3%)	5 (17%)
Much better	4 (14%)	5 (17%)
A little better	11 (38%)	11 (37%)
No change	12 (41%)	8 (27%)
A little worse	1 (3%)	1 (3%)
Much worse	0 (0%)	0 (0%)
Very much worse	0 (0%)	0 (0%)
Missing	15 (34%)	14 (32%)
TAU		
Very much better	0 (0%)	0 (0%)
Much better	0 (0%)	1 (3%)
A little better	4 (14%)	4 (12%)
No change	23/29 (79%)	22 (69%)
A little worse	1 (3%)	4 (12%)
Much worse	1 (3%)	1 (3%)
Very much worse	0 (0%)	0 (0%)
Missing data	13 (31%)	10 (24%)
*P*	0.001	< 0.001

PGI improvement for the two trial arms at the mid-treatment (6-week) and final (12-week) assessment points along with the significance level determined from a Mann–Whitney *U* test

#### Sexual health impact

The FSFI showed no statistically significant between group difference for either sexually active or non-active after 12 weeks. Both trial groups demonstrated an overall mean improvement 1.6 points for the control group compared to 2.6 for the Pelviva group (Table 4). No significant difference was seen between groups, whether sexually active or not in any individual domain (*P* > 0.12). No significant differences were seen at the 6-week mid-treatment assessment.

**Table 4:** FSFI and PISQ-IR results

Outcome	Arm	Baseline	Follow-up	Change	Treatment effect	*P*
FSFI-sexually active						
Overall score	TAU (*N* = 18)	25.3 (6.0)	26.9(5.0)	1.6 (2.8)		
	Pelviva (*N* = 22)	25.1 (6.9)	27.7(7.0)	2.6 (5.2)	0.98 (− 1.79 − 3.75)	0.48
FSFI score—Inactive						
	TAU (*N* = 5)	2.6 (1.5)	2.5 (1.0)	− 0.1 (1.1)		
	Pelviva (*N* = 4)	1.8 (1.2)	2.2 (1.2)	0.4 (0.9)	0.16 (− 1.56 − 1.87)	0.82
PISQ-IR Sexually active						
Overall score	TAU (*N* = 21)	2.9 (0.4)	3.0 (0.5)	0.0 (0.3)		
	Pelviva (*N* = 20)	2.9 (0.4)	3.1 (0.4)	0.2 (0.4)	0.21 (0.02 − 0.40)	0.032
Desire	TAU (*N* = 21)	2.9 (0.7)	2.9 (0.8)	− 0.0 (0.4)		
	Pelviva (*N* = 20)	3.1 (0.7)	3.2 (0.6)	0.1 (0.6)	0.13 (− 0.18 − 0.44)	0.41
Arousal/orgasm	TAU (*N* = 21)	3.3 (0.7)	3.5 (0.7)	0.2 (0.3)		
	Pelviva (*N* = 20)	3.5 (0.7)	3.8 (0.8)	0.3 (0.7)	0.15 (− 0.17 − 0.48)	0.35
Condition specific	TAU (*N* = 21)	1.8 (0.9)	1.7 (0.8)	− 0.1 (0.7)		
	Pelviva (*N* = 20)	1.7 (0.9)	1.5 (0.6)	− 0.2 (0.8)	− 0.21 (− 0.57 − 0.14)	0.23
Global quality	TAU (*N* = 21)	3.2 (1.0)	3.4 (1.1)	0.2 (0.6)		
	Pelviva (*N* = 20)	2.9 (0.9)	3.5 (0.9)	0.6 (1.0)	0.36 (− 0.12 − 0.84)	0.14
Condition impact	TAU (*N* = 21)	3.1 (0.9)	3.2 (0.8)	0.1 (0.6)		
	Pelviva (*N* = 20)	2.9 (1.0)	3.4 (0.7)	0.5 (0.8)	0.39 (0.05 − 0.74)	0.027
PISQ-IR sexually inactive						
Partner-related	TAU (*N* = 4)	3.0 (1.4)	2.8 (1.1)	− 0.2 (0.5)		
	Pelviva (*N* = 6)	2.8 (2.0)	2.8 (2.0)	0.0 (0.0)	0.18 (− 0.43 − 0.78)	0.49
Condition specific	TAU (*N* = 4)	3.9 (0.3)	3.5 (0.4)	− 0.4 (0.4)		
	Pelviva (*N* = 6)	3.3 (0.8)	3.7 (0.4)	0.3 (0.5)	0.40 (− 0.10 − 0.89)	0.096
Quality	TAU (*N* = 4)	2.0 (1.2)	2.2 (1.2)	0.2 (0.8)		
	Pelviva (*N* = 6)	2.5 (0.0)	2.5 (1.2)	0.0 (1.2)	− 0.10 (− 2.11 − 1.91)	0.90
Condition impact	TAU (*N* = 4)	3.9 (0.3)	3.5 (0.4)	− 0.4 (0.4)		
	Pelviva (*N* = 6)	3.3 (0.8)	3.7 (0.4)	0.3 (0.5)	0.40 (− 0.10 − 0.89)	0.096

Results for FSFI and PISQ-IR total score in sexually active and inactive women at the primary 12-week assessment point. Values are mean (SD). The effect size is the difference in outcome between arms adjusted for baseline and type of incontinence with 95% CI and associated significance level

The PISQ-IR followed a similar trend, though between group comparison in overall improvement reached statistical significance (*P* = 0.032). This seemed to be related to overall impact of the condition (*P* = 0.027) rather than any specific aspect of sexual activity i.e. desire, arousal/orgasm, condition specific or global quality though global quality showed almost as big an effect size as impact (Table 4). These improvements were not reported at the 6-week assessment.

#### Adverse events

Three adverse events were reported for the Pelviva group only. Two related to discomfort when using the device and one related to post-menopausal bleeding considered not be related to the device.

#### Effect of participant age

Stratifying the participants by age, those aged < 50 showed a stronger treatment response of − 2.59 points (95% CI − 4.65 to − 0.53) on the primary ICIQ outcome compared to those aged ≥ 50 who showed a − 0.60 point (− 3.53 to − 2.34) treatment effect. However, a formal interaction test did not show that this difference was statistically significant (*P* = 0.31).

## Discussion

### General findings

This study took place in a regular GP environment with outcome data collected on-line and in the privacy of the participants own home, a pragmatic ‘real world’ study. It was anticipated normal GP care at recruitment would be multi-faceted, but in practice, only 21–27% received pelvic floor exercise advice from the GP or a nurse and the remainder received little or no intervention. This does not correspond with the NICE (2019) guidelines [10] which recommend supervised pelvic floor muscle exercises for at least 3 months for stress urinary incontinence and at least 6 weeks of bladder retraining for urgency urinary incontinence.

Interestingly, the extent of intervention remained the same during the study for the Pelviva group, but the TAU group reported an increase in pelvic floor exercise from 27 to 64%. It would seem the Pelviva group did not perceive the need to change current intervention whilst the TAU group appeared more motivated to undertake exercise.

Despite differences in exercise rates, a significant improvement in the primary outcome-overall quality of life was observed between the Pelviva and control group (three-point compared to one point, respectively). This corroborates with the results from the previous study [three-point, 45% improvement vs one-point, 10% for ICIQ-UI (*P* = 0.014)] [9]. The control groups in the previous [9] and current study also behaved similarly suggesting little difference between GP led intervention or unsupervised pelvic floor exercises.

Improvements in the Pelviva group (1.6 points) were evident at 6 weeks suggesting improvement was cumulative over 12 weeks. It is not possible to determine if further improvements would have occurred if treatment continued beyond 12 weeks due to COVID-19 restrictions. Many clinical studies use 12 weeks as the optimum length of treatment though some do use a longer treatment intervention phase of up to 6 months [16].

QoL improvements in the Pelviva group were accompanied by reports of a global impression of improvement. Despite many women describing their incontinence as normal or mild when they started treatment, 17% of women in the Pelviva group described themselves as feeling very much better and a further 54% a little or much better after 12 weeks. Such improvements were not seen in the control group. It is not possible to determine why the Pelviva group felt better but women described their incontinence as interfering less in their lives.

Reduction of impact due to Pelviva also translated through to sexual health. The psychological effects of incontinence can have a considerable impact on quality of life. Women often report low self-esteem, mood changes and feelings of helplessness and even anxiety and depression [17, 18]. It is possible Pelviva offered women hope of improvement and this in turn reduced the psychological impact. It is also possible there was a quantitative improvement in volume of urine loss. It was the intention to monitor this using the 1-h pad test but due to COVID-19 this was not possible.

In terms of age, a formal interaction test for a difference in Pelviva effect was not significant (*P* = 0.31), so this study cannot conclude that the treatment has different effects in younger and older women. The Pelviva effect, however, seemed to be larger in the younger women, and whilst this could be a chance finding it should be considered further.

### Real world study and impact of COVID-19

A large number of women 13,052 were approached by text messaging to participate in this trial and 226 women (1.7%) responded. This response rate corresponds with Plante et al. [19] who approached 6896 American citizens and 116 (1.7%) people expressed an interest. Plante et al. also described a 10% recruitment rate that was lower than the 86 women (38%) in the current study. This could be due to the GP-led approach adopted for the current study compared to an investigator led recruitment suggesting a real-world GP approach gives greater assurance and confidence to patients and improves recruitment into clinical trials.

More women withdrew in the Pelviva group compared to TAU though non-responders were the same for both groups. Women who withdrew cited challenges on time and other commitments that may have been due to additional responsibilities and the impact of COVID-19. Excluding non-responders, adherence to Pelviva was 61%. Pelviva has been designed to be used in the privacy of the home so even in a COVID-19 situation self-treatment could continue demonstrating the considerable benefit of such a treatment approach.

### Limitations of the study

The study was under-powered due to the impact of the COVID-19 situation. Furthermore, the 1-h pad test could not be completed post-treatment as originally planned. Despite these limitations, the results from the first pad test showed the majority of women had apparently mild problems whilst responses for ICIQ covered the whole range of the scale (0–21) except for the very low end. The correlation between ICIQ and pad test responses was rather weak ($\rho$ = 0.35 for ICIQ total and 0.19 for amount). This would suggest that the 1-h test is not correlated with patients’ daily activities, has poor-to-moderate sensitivity and questions the validity of adopting the test in a Primary Care population with a self-administered treatment and real-world evaluation. In addition, it was not possible to blind patients to treatment group as the study was deliberately designed to test Pelviva against current GP practice and, therefore, not to incorporate a sham group.

## Conclusion

This study has demonstrated that Pelviva is a successful adjunct to GP prescribed care for the treatment of female urinary incontinence. Statistically significant superior results were seen in relation to overall quality of life, interference with life and impact on sexual health. These results were observed despite early termination of this study due to COVID-19 and a considerably reduced sample size. The study also highlighted some limitations of the 1-h pad test when assessing severity of urine loss.
